# Bedside Transcranial Doppler as a Dynamic Warning Tool for Rapidly Evolving Intracranial Hypertension in Pneumococcal Meningitis

**DOI:** 10.1002/ccr3.73733

**Published:** 2026-10-13

**Authors:** Mahmoud Ahmed, Victoria Collins, Andrea Lavinio

**Affiliations:** ^1^ Department of Intensive Care Medicine and Anaesthesia Cambridge University Hospitals NHS Foundation Trust Cambridge UK; ^2^ Perioperative, Acute, Critical Care and Emergency Medicine (PACE) Section, Department of Medicine University of Cambridge, Cambridge Biomedical Campus Cambridge UK

**Keywords:** bacterial meningitis, cerebrospinal fluid, critical care, hydrocephalus, intracranial hypertension, transcranial Doppler ultrasonography

## Abstract

In severe bacterial meningitis, an initially non‐critical or technically unreliable lumbar puncture opening pressure should not reassure when hydrocephalus and coma persist. Bedside transcranial Doppler cannot measure intracranial pressure, but bilateral near‐absent diastolic flow may provide an urgent warning signal prompting repeat assessment and cerebrospinal fluid diversion.

## 1 Introduction

*Streptococcus pneumoniae* remains a leading cause of community‐acquired bacterial meningitis in adults and is associated with high mortality and neurological morbidity [1, 2, 3]. Intracranial hypertension is an important pathway to secondary cerebral injury, but its recognition can be difficult when neurological examination is limited by coma, intubation, or sedation.

Lumbar puncture (LP) with opening pressure measurement is a standard bedside method for assessing cerebrospinal fluid (CSF) pressure. However, manometry depends on free communication between the CSF compartment, the spinal needle and the manometer. In severe bacterial meningitis, dense inflammatory, proteinaceous CSF may impair flow through the needle, making a single pressure value technically unreliable. Intracranial pressure (ICP) may also change rapidly as inflammation, hydrocephalus and impaired CSF resorption evolve [4].

Transcranial Doppler ultrasonography (TCD) is a non‐invasive bedside method for assessing cerebral haemodynamics [5]. TCD does not directly measure ICP, but marked loss of diastolic flow can signal severely compromised cerebral perfusion when interpreted in the appropriate clinical context. We describe a case in which bedside TCD acted as an escalation signal after technically unreliable LP manometry in pneumococcal meningitis.

## 2 Case History and Examination

A previously independent woman in her 70s presented to the emergency department via ambulance with fever, vomiting and markedly reduced consciousness. Her husband described 1 week of coryzal symptoms followed by 2 days of right‐sided headache, otalgia and ear discharge, with rapid deterioration in alertness during the day before admission (Table 1).

**TABLE 1:** Clinical timeline.

Timepoint	Clinical event and decision
Day 1: ED arrival	GCS 6, fever, vomiting and hypertension. CT showed communicating hydrocephalus, transependymal CSF migration and right mastoid/middle‐ear opacification, without herniation.
Day 1: first LP	LP after intubation yielded thick, turbid CSF. Opening pressure 23 cmH_2_O with closing pressure approaching zero; measurement considered technically unreliable.
Day 1: NCCU admission	Antimicrobial therapy continued; dexamethasone commenced after LP. Neurosurgical review supported close reassessment rather than immediate CSF diversion.
Approximately 5 h later	Bilateral TCD showed preserved systolic flow with markedly attenuated, near‐absent diastolic flow, prompting urgent reassessment.
Immediately after TCD	Repeat LP opening pressure 42 cmH_2_O; 40 mL turbid CSF drained therapeutically.
Day 2	Right frontal EVD inserted; turbid CSF under high pressure on first pass. Right tympanostomy tube inserted.
Day 3	Right cortical mastoidectomy; two tegmen dehiscences identified.
Days 5–7	CT showed mild reduction in ventricular size. EVD clamped day 5, removed day 6; extubated day 7.
Days 12–13	Transferred to neurology ward day 12; GCS 15 documented day 13.
Follow‐up	Specialist neurorehabilitation with improving mobility and engagement, but persistent short‐term memory and concentration impairment.

Abbreviations: CSF, cerebrospinal fluid; CT, computed tomography; ED, emergency department; EVD, external ventricular drain; GCS, Glasgow Coma Scale; LP, lumbar puncture; NCCU, Neurosciences and Trauma Critical Care Unit; TCD, transcranial Doppler.

On arrival her Glasgow Coma Scale score was 6 (E4M1V1). She was febrile at 39°C, tachycardic at 119 beats/min and hypertensive at 164/74 mmHg. Oxygen saturation was 98% on 15 L/min oxygen via a non‐rebreathing mask. There was no rash. Arterial blood gas analysis on high‐flow oxygen showed respiratory alkalosis (pH 7.59, pCO_2_ 3.3 kPa) and lactate 3.71 mmol/L; blood glucose was 18 mmol/L.

Computed tomography (CT) of the head showed communicating hydrocephalus with periventricular lucency consistent with transependymal CSF migration, together with opacification of the right mastoid air cells and middle ear. There was no midline shift or cortical herniation (Figure 1). Empirical ceftriaxone, amoxicillin, and aciclovir were commenced for suspected community‐acquired meningitis.

**FIGURE 1:**
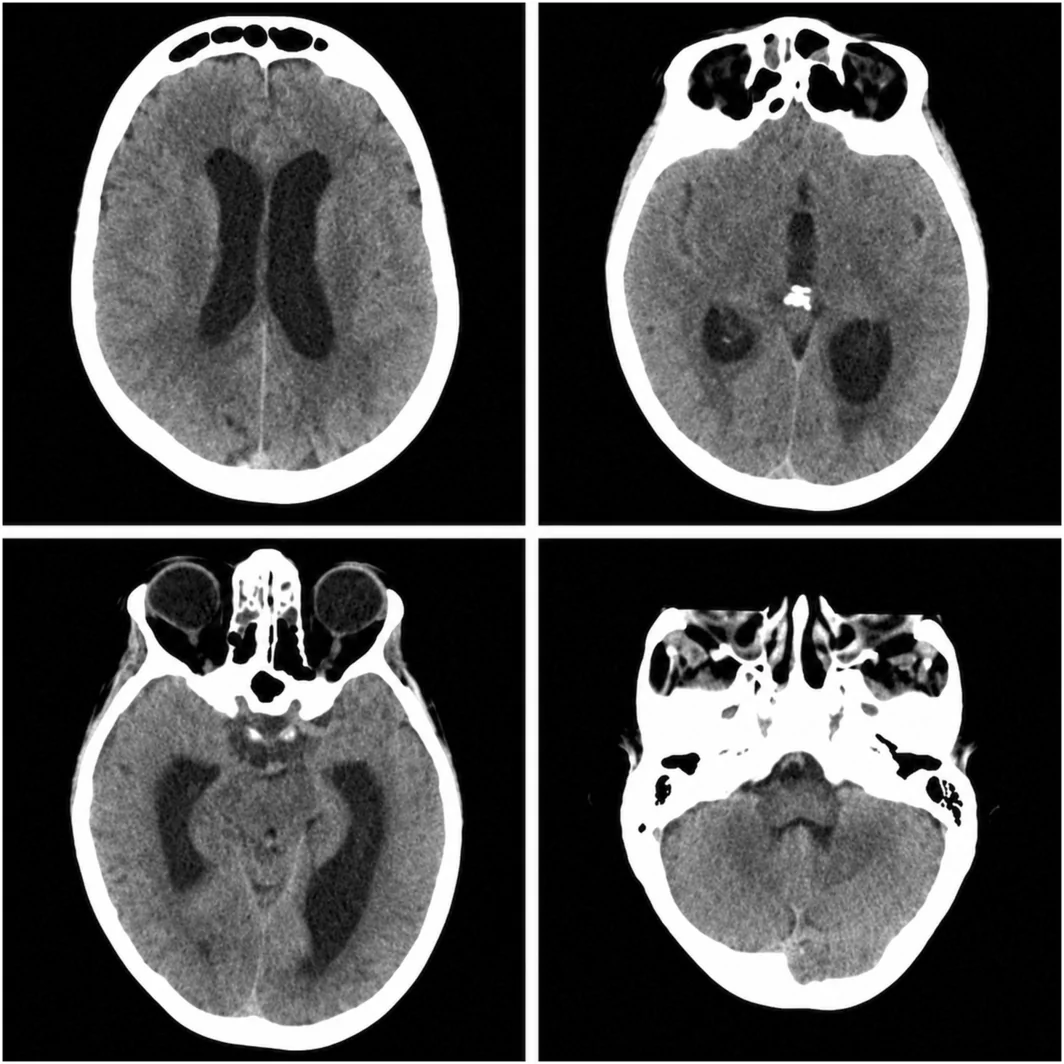
Admission CT head. Axial slices demonstrate communicating hydrocephalus with periventricular lucency consistent with transependymal CSF migration and opacification of the right mastoid air cells and middle ear, consistent with the otogenic source of infection. No midline shift or cortical herniation.

Because of ongoing clinical deterioration and vomiting, the patient was intubated for airway protection. LP was then performed while she was intubated, ventilated and sedated. The CSF was thick, turbid, blood‐stained and proteinaceous. The manometer column could not be filled adequately; an opening pressure of 23 cmH_2_O (17 mmHg) was recorded on reattempt, with a closing pressure approaching zero. Both values were considered technically unreliable at the time because adequate CSF flow through the needle could not be established.

## 3 Investigations, Differential Diagnosis and Treatment

CSF analysis showed 679 white cells/μL (671 polymorphs and 8 lymphocytes), red blood cells 313 cells/μL, protein 10.42 g/L (reference range 0.15–0.45 g/L), glucose < 0.5 mmol/L and lactate 30.8 mmol/L (reference range 1.1–2.8 mmol/L) (Table 2). Gram stain showed Gram‐positive cocci in pairs and chains; pneumococcal latex agglutination was positive, and blood and CSF cultures subsequently confirmed *Streptococcus pneumoniae* .

**TABLE 2:** Serial CSF parameters.

Parameter	Day 1—first LP	Day 2—EVD sample	Day 6—EVD removal	Day 17
Appearance	Turbid	Turbid	Clear	Clear
Protein (g/L; ref. 0.15–0.45)	10.42	—	0.49	2.24
Glucose (mmol/L)	< 0.5	—	3.5	2.4
Lactate (mmol/L; ref. 1.1–2.8)	30.8	—	—	—
Polymorphs (cells/μL)	671	461	13	< 5
Lymphocytes (cells/μL)	8	3	3	39
RBC (cells/μL)	313	580	2	290
Opening pressure	23 cmH_2_O (17 mmHg) – technically unreliable	High pressure at EVD insertion	—	—

*Note:* Markedly abnormal admission CSF protein and lactate support a dense inflammatory burden and help explain why manometry could be technically unreliable. The second LP CSF was drained therapeutically and discarded, so no laboratory sample was sent.

Dexamethasone 6.6 mg four times daily was started immediately after LP. Neurosurgical review was undertaken. Because the hydrocephalus appeared communicating, there was no focal herniation and ventricular catheterization during active infection carried risk, initial management was conservative with planned close reassessment. The technically uncertain LP pressure contributed to clinical equipoise regarding immediate CSF diversion.

The differential diagnosis for persistent coma and neurological deterioration included progressive communicating hydrocephalus due to impaired CSF resorption, cerebral oedema from meningeal inflammation, meningitis‐associated vasculopathy or vasospasm, cerebral venous sinus thrombosis, seizure or non‐convulsive status epilepticus, and sedative or metabolic contributors. CT evidence of hydrocephalus, severely abnormal CSF biochemistry, and discordance between the imaging and the technically uncertain opening pressure made evolving intracranial hypertension a major concern.

Approximately 5 h later, bedside TCD was performed using a GE Venue Go point‐of‐care ultrasound system with a 3Sc sector probe (GE HealthCare, Chicago, Illinois, USA) through the bilateral transtemporal acoustic windows. The middle cerebral arteries were insonated bilaterally, and the spectral waveforms were assessed for preservation of systolic forward flow and the degree of diastolic flow.

On transcranial color‐coded duplex imaging, the intracranial arterial color signal was clearly visible during systole but markedly diminished or disappeared during diastole, producing a striking “peek‐a‐boo” appearance; on spectral Doppler this corresponded to preserved systolic forward flow with markedly attenuated, near‐absent diastolic flow bilaterally. The term “peek‐a‐boo” is used here as descriptive bedside shorthand rather than as a standardized Doppler sign. Figure 2 provides an original physiological schematic of this waveform pattern and is not the patient's recorded Doppler spectrum.

**FIGURE 2:**
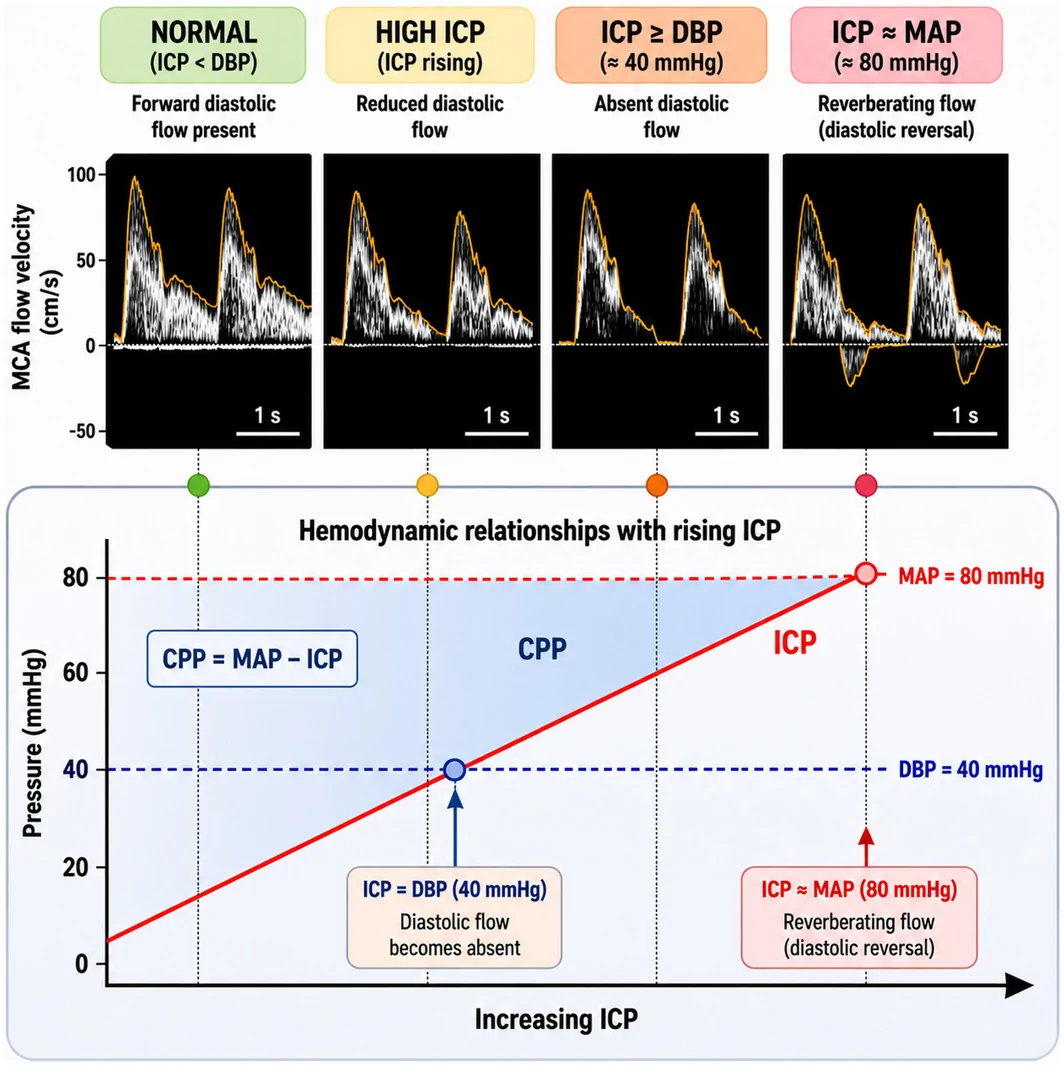
Schematic representation of transcranial Doppler waveform changes with rising intracranial pressure. As intracranial pressure rises, diastolic flow is affected first because cerebral perfusion pressure is lowest during diastole. Preserved systolic forward flow with markedly attenuated or near‐absent diastolic flow is a warning pattern consistent with critically impaired cerebral perfusion when interpreted alongside clinical and radiological evidence of hydrocephalus. This figure is an original physiological schematic and not the patient's Doppler spectrum.

TCD did not establish the diagnosis of intracranial hypertension. Rather, the bilateral waveform abnormality acted as an escalation signal that prompted immediate reassessment. Immediate repeat LP measured an opening pressure of 42 cmH_2_O (30 mmHg), confirming critical intracranial hypertension. Forty milliliters of turbid CSF were drained therapeutically. Following urgent neurosurgical discussion and CT brain volumetry, a right frontal external ventricular drain (EVD) was inserted on day 2. Turbid CSF was encountered under high pressure on the first pass, and the EVD was set at 15 cmH_2_O.

Source control was achieved with right tympanostomy tube insertion on day 2 and right cortical mastoidectomy on day 3. The mastoidectomy identified two tegmen dehiscences, providing the likely route of intracranial infection. Ceftriaxone was continued as definitive therapy for 4 weeks. CT venography later demonstrated partially occlusive left transverse sinus thrombosis and a small right transverse sinus filling defect, consistent with septic lateral sinus thrombosis and cerebral venous sinus thrombosis. Anticoagulation was commenced with low molecular weight heparin and subsequently transitioned to rivaroxaban after multidisciplinary hematology review.

## 4 Outcome and Follow‐Up

The patient survived despite several poor prognostic features at presentation, including GCS 6, communicating hydrocephalus, multifocal intracranial disease and cerebral venous sinus thrombosis. CT on day 5 showed mild reduction in ventricular size; the EVD was clamped on day 5 and removed on day 6. She was extubated on day 7 and transferred to the neurology ward on day 12. By day 13, GCS was documented as 15 (E4V5M6), with improving alertness and speech.

Subsequent recovery included fluctuating consciousness, sleep–wake cycle disturbance, dysphonia, dysphagia requiring nasogastric feeding, and cognitive‐linguistic impairment. She was transferred to specialist neurorehabilitation. At follow‐up she was improving in mobility, vocalization and engagement with therapy, but continued to experience marked short‐term memory and concentration difficulties, with intermittent confusion. Discharge home was being planned.

## 5 Discussion

This case illustrates a practical problem in severe bacterial meningitis: a single LP opening pressure can be misleading when both measurement reliability and intracranial physiology are changing. The initial opening pressure of 23 cmH_2_O could have appeared only modestly elevated, but the inability to fill the manometer and the near‐zero closing pressure indicated unreliable flow dynamics. The repeat pressure of 42 cmH_2_O 5 h later is consistent with both the technical unreliability of the first measurement and genuine dynamic ICP escalation.

The case also shows why radiological‐clinical discordance should trigger serial reassessment. CT already demonstrated communicating hydrocephalus and transependymal CSF migration. In that context, persistent coma should not be reassured by a technically difficult pressure measurement. Clinical observations in bacterial meningitis support the concept that intracranial complications may evolve as inflammatory exudate accumulates, CSF resorption becomes impaired, and cerebral haemodynamics deteriorate [4].

Dexamethasone was appropriately commenced after LP and therefore could not have influenced the initial recorded pressure. Its use does not remove the need for ongoing ICP surveillance. Steroid therapy may attenuate inflammatory injury in pneumococcal meningitis [6], but would not be expected to rapidly reverse mechanical impairment of CSF resorption from dense proteinaceous exudate.

TCD should be interpreted cautiously. It is operator‐dependent; adequate transtemporal acoustic windows may not be obtainable in all patients, and waveform morphology is influenced by arterial pressure, PaCO_2_, cerebrovascular resistance and other physiological factors. It is not a substitute for direct CSF pressure measurement or invasive ICP monitoring and cannot provide an exact ICP measurement [7]. Other bedside sonographic approaches, particularly optic nerve sheath diameter ultrasonography, may provide additional indirect evidence of intracranial hypertension, although these techniques are likewise operator‐dependent and should be integrated with the clinical examination, imaging and direct pressure measurements where available [5]. The physiological basis of the warning pattern is that cerebral perfusion pressure equals mean arterial pressure minus ICP. As downstream pressure rises, forward flow is lost first during diastole, when the driving pressure is lowest. Progressive ICP elevation may produce reduced diastolic velocity, near‐absent diastolic flow, and eventually oscillating or reverberating flow associated with cerebral circulatory arrest [5]. In this patient, the bilateral waveform abnormality was meaningful because it was integrated with persistent coma, CT‐confirmed hydrocephalus, highly abnormal CSF, and the earlier technically unreliable LP.

Published TCD studies in bacterial meningitis have focused mainly on cerebral blood flow abnormalities, vasculopathy and vasospasm rather than dynamic escalation after unreliable LP manometry [8]. Invasive ICP monitoring and CSF diversion have been described in severe bacterial meningitis, but evidence remains limited and non‐randomized [9, 10]. This report should therefore be read as an educational case and reasoning pathway, not as evidence of diagnostic accuracy or a general treatment threshold.

The central lesson is reproducibility. Urgent bedside studies that influence management should be captured whenever possible. Saved TCD spectra, bilateral velocities, pulsatility indices, arterial pressure, PaCO_2_, ventilator settings, sedation and vasoactive therapy would have allowed independent review and stronger comparison over time. Their absence is a major limitation of this report. Repeat TCD after therapeutic CSF drainage or following EVD insertion was not available, so reversal of the waveform abnormality after ICP reduction could not be demonstrated; such paired assessment would have provided stronger physiological support for the proposed interpretation.

## 6 Conclusion

In comatose bacterial meningitis with hydrocephalus, an apparently non‐critical LP opening pressure should not reassure when manometry is technically difficult or CSF flow is poor. Bedside TCD cannot measure ICP, but a severely abnormal bilateral waveform may provide an urgent, repeatable haemodynamic warning that should prompt integration with imaging and clinical findings, repeat measurement and escalation to CSF diversion when appropriate.

## Author Contributions

**Mahmoud Ahmed:** writing – review and editing. **Victoria Collins:** writing – review and editing. **Andrea Lavinio:** conceptualization, writing – original draft, writing – review and editing.

## Funding

The authors have nothing to report.

## Disclosure

Patient Perspective: The patient's husband described the neurocritical care admission as traumatic and expressed gratitude to the teams involved. He hoped that sharing the case would improve recognition and treatment of severe meningitis, while also encouraging clinicians to combine technical expertise with sensitive communication to families during life‐threatening illness. His perspective is included with permission.

## Ethics Statement

This report describes clinical care rather than research. Under UK Health Research Authority guidance, formal Research Ethics Committee or Institutional Review Board approval was not required. Institutional permission for publication has been obtained.

## Consent

Written informed consent for publication of the patient's clinical details and images was obtained from the patient's husband because the patient lacked capacity to provide consent at the time. The signatory's relationship to the patient and the reason for representative consent are documented on the signed consent form. The authors confirm that the consent process covered publication of clinical information and images in a medical journal and online, and that the patient representative had the opportunity to review and comment on the manuscript.

## Conflicts of Interest

A.L. reports consultancy fees and an educational grant from Becton, Dickinson and Company and Masimo; he holds equity in and serves as a consultant for Neuron Guard; and he is a Section Editor at the Journal of Critical Care. M.A. and V.C. declare no competing interests.

## Data Availability

All clinically relevant information is included in this article. No patient‐level dataset is publicly available because of confidentiality.
